# MiR-101 Targets the EZH2/Wnt/β-Catenin the Pathway to Promote the Osteogenic Differentiation of Human Bone Marrow-Derived Mesenchymal Stem Cells

**DOI:** 10.1038/srep36988

**Published:** 2016-11-15

**Authors:** Hongrui Wang, Yake Meng, Quanjun Cui, Fujun Qin, Haisong Yang, Yu Chen, Yajun Cheng, Jiangang Shi, Yongfei Guo

**Affiliations:** 1 Departmentof Orthopaedics, Changzheng Hospital, The Second Military Medical University of China, 415 Fengyang Road, Shanghai 200003, P.R. China.; 2 Department of Orthopaedics,Changhai Hospital, The Second Military Medical University of China, 168 Changhai Road, Shanghai 200433, P.R. China; 3 Department of Orthopedic Surgery,University of Virginia, 400 Ray C. Hunt Drive, Charlottesville, VA 22903, USA; 4 Department of Pathology, University of Virginia, Charlottesville VA 22908, USA

## Abstract

Mounting evidence indicates that microRNAs (miRNAs) are involved in multiple processes of osteogenic differentiation. MicroRNA-101 (miR-101), identified as a tumor suppressor, has been implicated in the pathogenesis of several types of cancer. However, the expression of miR-101 and its roles in the osteogenic differentiation of human bone marrow-derived mesenchymal stem cells (hBMSCs) remain unclear. We found that the miR-101 expression level was significantly increased during the osteogenic differentiation of hBMSCs. MiR-101 depletion suppressed osteogenic differentiation, whereas the overexpression of miR-101 was sufficient to promote this process. We further demonstrated that enhancer of zeste homolog 2 (EZH2) was a target gene of miR-101. EZH2 overexpression and depletion reversed the promoting or suppressing effect of osteogenic differentiation of hBMSCs, respectively, caused by miR-101. In addition, we showed that miR-101 overexpression promoted the expression of Wnt genes, resulting in the activation of the Wnt/β-catenin signaling pathway by targeting EZH2, while the activity of β-catenin and the Wnt/β-catenin signaling pathway was inhibited by ICG-001, a β-Catenin inhibitor, which reversed the promoting effect of miR-101. Finally, miR-101 also promotes *in vivo* bone formation by hBMSCs. Collectively, these data suggest that miR-101 is induced by osteogenic stimuli and promotes osteogenic differentiation at least partly by targeting the EZH2/Wnt/β-Catenin signaling pathway.

Bone marrow-derived mesenchymalstem cells (MSCs) are multipotent stromal cells that have the capability to differentiate into a variety of cell types including osteoblasts^1^. Osteoblasts, as a specialized population of cells, play an important role in bone formation by secreting bone matrix proteins^2^. Recently, the regulatory mechanism of osteogenic differentiation from MSCs has been investigated and might have significant clinical potential in stem cell-based therapeutic strategies for bone loss diseases, such as osteoporosis or to regenerate damaged bone^3^. However, the underlying molecular mechanisms of MSC osteogenic differentiation still remain unclear. Therefore, in order to develop more effective therapy for bone loss diseases, itis important to uncover the molecular mechanism for MSC osteogenic differentiation.

MiRNAs are small and evolutionarily conserved noncoding RNAs that play a pivotal role in various biological processes, including cellular differentiation, proliferation, apoptosis and tissue development via regulating the expression of target genes by binding their mRNA 3^′^-untranslated regions^4^. Increasing evidence shows that posttranslational repression of gene expression mediated by miRNAs, plays an important role in regulating the osteogenic differentiation of MSCs^5^,^6^. For example, miR-96, miR-124, miR-199a, miR-100, miR-138, miR-204 and miR-20a have been reported to regulate osteoblast differentiation by targeting various osteoblast genes^5^,^7^,^8^,^9^,^10^,^11^. Further investigation is required to clarify whether the osteogenic differentiation is regulated by other miRNAs. MiR-101, frequently silenced in human cancers, has been shown to have fundamental roles in diverse cellular processes, especially in the control of proliferation and differentiation^12^,^13^,^14^,^15^. Notably, Li *et al.*^16^ reported that miR-101 may promote the osteogenic differentiation of periodontal ligament cells by targeting periodontal ligament associated protein-1 (PLAP-1/aspirin). However, the roles of miR-101 in regulating the osteogenic differentiation of hBMSCs remain unknown.

Recent evidence has reported that EZH2 inhibits the osteogenic differentiation of MSCs by repressing the Wnt gene, which leads to the inactivation of Wnt/β-cateninsignaling^17^. Meanwhile, EZH2, which plays an important biological role, has been reported to be a target gene of miR-101 in various cells. For example, miR-101 inhibited human hepatocellular carcinoma progression by targeting EZH2^18^. Therefore, the aims of the present study were to explore the expression and role of miR-101 in the osteogenic differentiation of hBMSCs, to investigate whether miR-101 regulates osteogenic differentiation by targeting EZH2 and to validate the regulatory relationship betweenmiR-101 and the Wnt/β-Catenin signaling pathway.

## Results

### Expression of miR-101 is increased during osteogenic differentiation of hBMSCs

**Figure 1:**
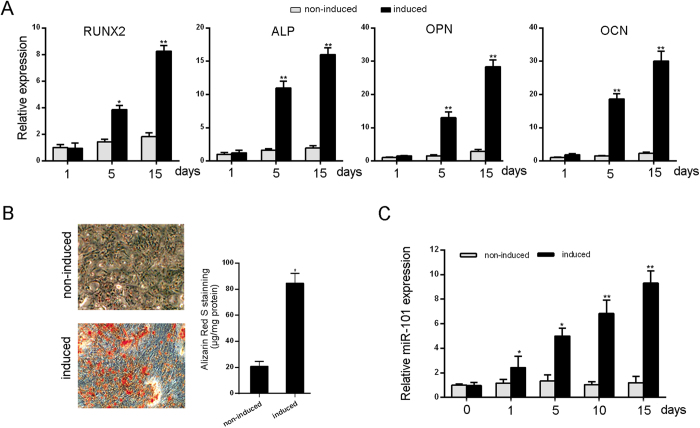
miR-101 is upregulated during the osteogenic differentiation of hBMSCs. (**A**) The mRNA expression levels of the osteoblastic marker genes RUNX2, ALP, and OPN were analyzed by qRT-PCR at day 1, 10 and 15, with β-actin as a control. (**B**) Alizarin Red staining was measured at day 15 and quantification is shown at right. (**C**) The relative expression of miR-101 was measured by qRT-PCR. U6 was used as a control. All of the data are presented as the mean ± SD. *p < 0.05; **p < 0.01versus the non-induced group.

To investigate whether miR-101 is involved in regulating osteogenic differentiation, hBMSCs were cultured in osteogenic differentiation medium. As shown in Fig. 1A, the expression levels of osteoblastic marker genes including RUNX2, ALP, OPN and OCN were significantly increased. Correspondingly, Alizarin Red staining experiments confirmed the osteoblast phenotype (Fig. 1B). Next, the expression of miR-101 was examined at different time points during osteoblast differentiation. The results showed a gradual upregulation of the miR-101 expression during the osteogenic induction differentiation of hBMSCs (Fig. 1C), suggesting that miR-101 might be involved in regulating the osteogenic differentiation of hBMSCs.

### MiR-101 promotes the osteoblast differentiation of hBMSCs

**Figure 2:**
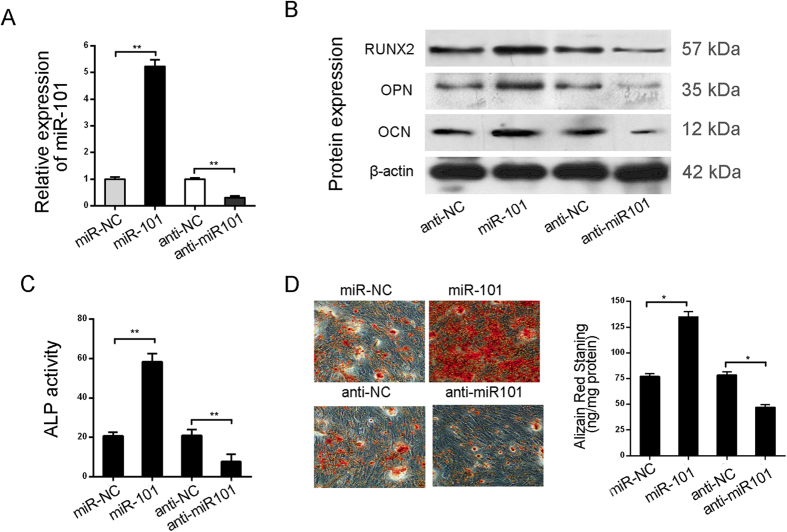
miR-101 promotes the osteogenic differentiation of hBMSCs. (**A**) miR-101 mRNA expression in hBMSCs infected by miR101-lentivirus or transfected with anti-miR101 was analyzed by qRT-PCR with U6 as a control before osteogenic differentiation. (**B**) The osteoblastic marker genes RUNX2, ALP, OPN and OCN were analyzed by Western Blot at day 15 of osteogenic differentiation with β-actin as a control. (**C**) ALP activity was measured at day 15. (**D**) Alizarin Red staining was measured at day 15 and quantification is shown at right. All of the data are expressed as the mean ± SD. *p < 0.05, **p < 0.01.

To identify the biological role of miR-101 in the regulation of hBMSC differentiation, cells were infected with miR-101lentivirusto establish stably miR-101-overexpressing hBMSCs or transfected with anti-miR-101oligonucleotideto inhibitmiR-101expression. The expression level of miR-101 was verified by qRT-PCR (Fig. 2A). In addition, miR-101 overexpression apparently promoted osteogenic differentiation, which was indicated by the up-regulated osteogenic marker genes RUNX2, ALP, OPN and OCN. In contrast, silencing of miR-101significantly inhibited osteogenic differentiation, accompanied by lower expression of the osteogenic marker genes (Fig. 2B). Furthermore, compared to the negative control, up-regulation of miR-101 induced ALP activity and the matrix mineralization level, while miR-101 silencing resulted in a decrease in their expression (Fig. 2C,D). Taken together, these results indicate that miR-101 plays a positive role in the regulation of the osteogenic differentiation of hBMSCs.

### MiR-101 directly targets EZH2 and inhibits its expression

**Figure 3:**
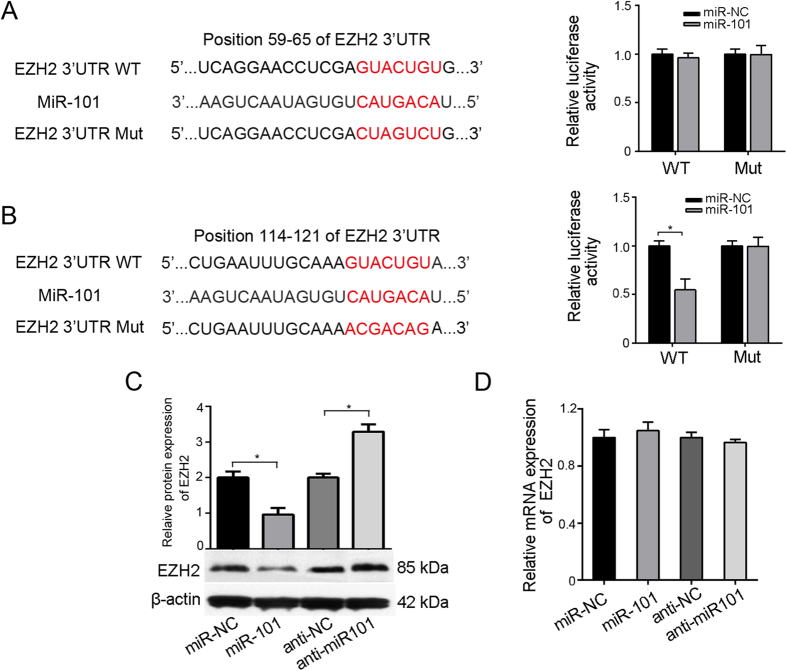
EZH2 is a target of miR-101. (**A**,**B**) Left: Schematic representation of the miR-101 site in theEZH2 3′-UTR (up: position 59–65; down: position 114–121);Right: The 3′-UTR reporter assay was carried out in hBMSCs infected with miR-101lentivirus or miR-NC lentivirus. The WT or Mut reporter plasmids were transfected with Lipo-2000. Luciferase assays were performed 48 h after transfection. Firefly luciferase activity was standardized to a Renilla luciferase control. (**C**) EZH2 protein expression and (**D**) mRNA level in hBMSCs infected with miR-101lentivirus or transfected with anti-miR-101 were measured after 48 h. All of the data are expressed as the mean ± SD. *p < 0.05, **p < 0.01.

In order to determine the target genes of miR-101, candidates were searched using theTargetScan6.2 (http://www.targetscan.org/) and miRBase (http://www.mirbase.org/) micoRNA databases. The results showed that miR-101 targets the EZH2 gene at the positions 59–65 and 114-121 (Both left panel of Fig. 3A,B). To analyze the relationship between miR-101 andEZH2, a luciferase reporter assay containing the wild-type (wt) or mutant (mut) miR-101 target sites in the EZH23′-UTRwas performed in HBMSCs. The results showed that the overexpression of miR-101 significantly decreased the luciferase activity of the EZH2wt-3′-UTRs (114-121) compared to miR-control transfected cells (Right panel; Fig. 3B), whereas it had no effect on the EZH2wt-3′-UTRs (59-65) (Right panel; Fig. 3A), indicating that miR-101 suppressed the expression of EZH2 by binding to target sites in their 3′-UTRs (114–121). Furthermore, Western blot analysis showed that overexpression of miR-101 substantially decreased the expression of EZH2, while anti-miR-101 transfection increased theEZH2 protein levels (Fig. 3C). Notably, the qRT-PCR analysis showed that neither miR-101 overexpression noranti-miR-101 transfection had significant effects on theEZH2 mRNA levels (Fig. 3D), suggesting that miR-101 specifically regulatedEZH2expression at the posttranscriptional level. These results support the bioinformatics predictions and indicate that EZH2 may be a direct target of miR-101.

### EZH2 mediates miR-101-regulated osteogenic differentiation of hBMSCs

**Figure 4:**
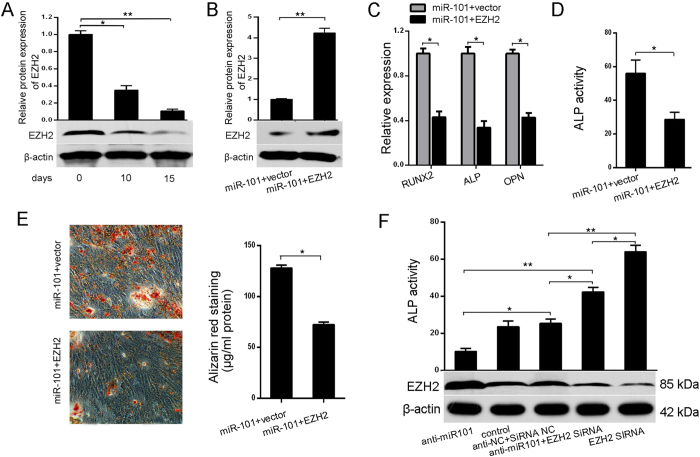
EZH2 mediates the miR-101-regulated osteogenic differentiation of hBMSCs. (**A**) EZH2expression was examined at different time points during osteoblast differentiation by Western blot. (**B**) The expression of EZH2 was assessed by Western blot. (**C**,**D**) EZH2 overexpression inhibits the expression of osteoblastic marker genes and reduces the ALP activity in miR-101-overexpressing cells. (**E**) Alizarin Red staining was measured and quantification is shown at right. (**F**) Up: ALP activity was measured; down: EZH2 expression was analyzed by Western blot. All of the data are expressed as the mean ± SD. *p < 0.05, **p < 0.01.

To ascertain the exact role of EZH2 in miR-101-regulated osteogenic differentiation, the expression of EZH2 was first examined at different time points during osteogenic differentiation. The immunoblotting results indicated a gradual down-regulation of EZH2 during osteogenic induction differentiation (Fig. 4A). Next, we evaluated whether EZH2 up-regulation was required for promoting the effect of miR-101 in regulating osteogenic differentiation. We restored the expression of EZH2 inmiR-101-overexpressing hBMSCs by transfecting the cells with EZH2 ORF constructs without 3′-UTRs (Fig. 4B), and qRT-PCR analyses showed that the expression of osteogenic marker genes, including RUNX2, ALP, and OPN, were suppressed by EZH2 rescue in the miR-101-overexpressing cells (Fig. 4C). The overexpression of EZH2 also significantly decreased ALP activity and matrix mineralization (Fig. 4D,E), suggesting that EZH2 overexpression impaired the effect ofmiR-101 in the osteogenic differentiation of hBMSCs. Co-transfection of EZH2-siRNA with anti-miR-101 almost completely blocked the negative effect of anti-miR-101 on ALP activity (Fig. 4F), and EZH2-siRNA significantly increased ALP activity, indicating enhanced osteoblastic differentiation. Taken together, these results suggest that EZH2isa functional target ofmiR-101.

### miR-101 promotes the osteogenic differentiation of hBMSCs by targeting EZH2 and regulating the Wnt/β-catenin pathway

**Figure 5:**
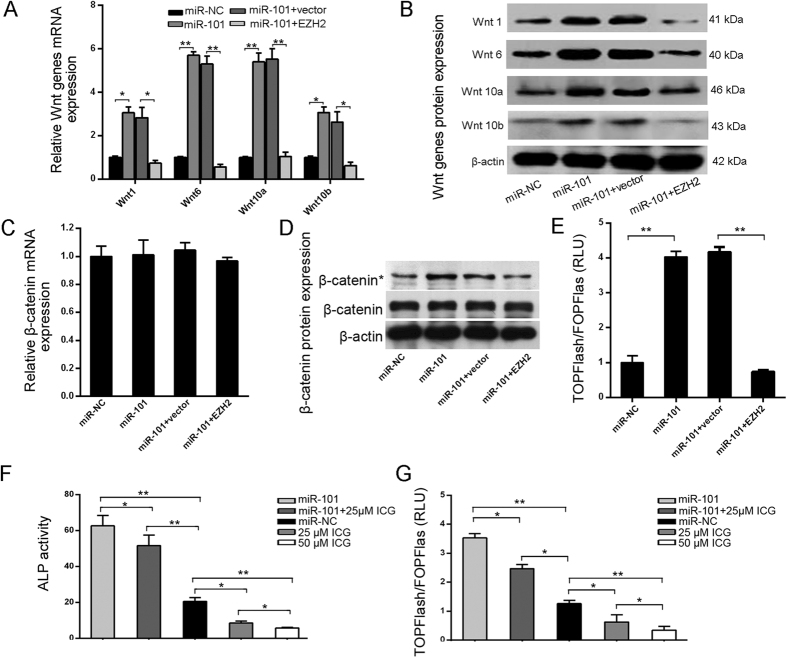
miR-101 regulates the osteogenic differentiation of hBMSCs by regulating the Wnt/β-catenin pathway. (**A**,**B**) Wnt gene mRNA and protein expression was analyzed by qRT-PCR and Western blot, respectively. (**C**,**D**) β-catenin and active β-catenin (β-catenin*) expression were analyzed by qRT-PCR and Western blot, respectively. (**E**) Luciferase activity of TOPFlash/FOPFlash. (**F**) ALP activity was measured in miRNA-101 overexpressing hBMSCs treated with ICG-001. (**G**) Luciferase activity of TOPFlash/FOPFlash in miRNA-101 overexpressing hBMSCs treated with ICG-001. All of the data are expressed as the mean ± SD. *p < 0.05, **p < 0.01.

The mechanism of how EZH2 inhibits osteogenic differentiation has not been clarified; the inactivation of Wnt/β-catenin signaling caused by EZH2 via repressing Wnt expression could explain this phenomenon to a certain extent^16^. To address these issues, the expression of Wnt genes and the activity of β-catenin were analyzed in the context of miR-101 overexpression ormiR-101 overexpression in conjunction with an EZH2 vector. QRT-PCR analyses showed that the expression of Wnt genes, including Wnt1, Wnt6, Wnt10a and Wnt10b, were up-regulated by miR-101 overexpression, and ectopic EZH2 expression eliminated the promoting effect of miR-101 overexpression on Wnt genes expression (Fig. 5A,B). Furthermore, we found that miR-101 and EZH2 overexpression did not change the expression of total β-catenin both at the mRNA and the protein levels, but miR-101 overexpression increased the expression of activated β-catenin (β-catenin*), and ectopic EZH2 expression blocked the promoting effect of miR-101 overexpression on activity of β-catenin (Fig. 5C,D). To demonstrate that the Wnt/β-catenin axis is responsible for miR-101-triggered osteogenic differentiation, the TOPFlash assay showed that miR-101 overexpression significantly increased the activity and ectopic EZH2 expression blocked the promoting effect of miR-101 overexpression on TOPFlash activity (Fig. 5E). These results showed that miR-101 activated the Wnt/β-catenin signaling pathway in hBMSCs by targeting EZH2. Finally, to pinpoint the role of the Wnt/β-catenin signaling pathway in miR-101-induced osteogenic differentiation of hBMSCs, ICG-001, an inhibitor of β-catenin, was used to inhibit the activity of β-catenin^18^. The results showed that ICG-001 can suppress the activity of ALP and TOPFlash in hBMSCs and miR-101-overexpressing hBMSCs, demonstrating that the promoting effect of the Wnt/β-catenin pathway activity and osteogenic differentiation of hBMSCs caused by miR-101 can be blocked by ICG-001 (Fig. 5F,G). Collectively, these data confirm that miR-101 promotes the osteogenic differentiation of hBMSCs by targeting EZH2 and regulating the Wnt/β-catenin pathway.

### miR-101 promotes *in vivo* bone formation by hBMSCs

**Figure 6:**
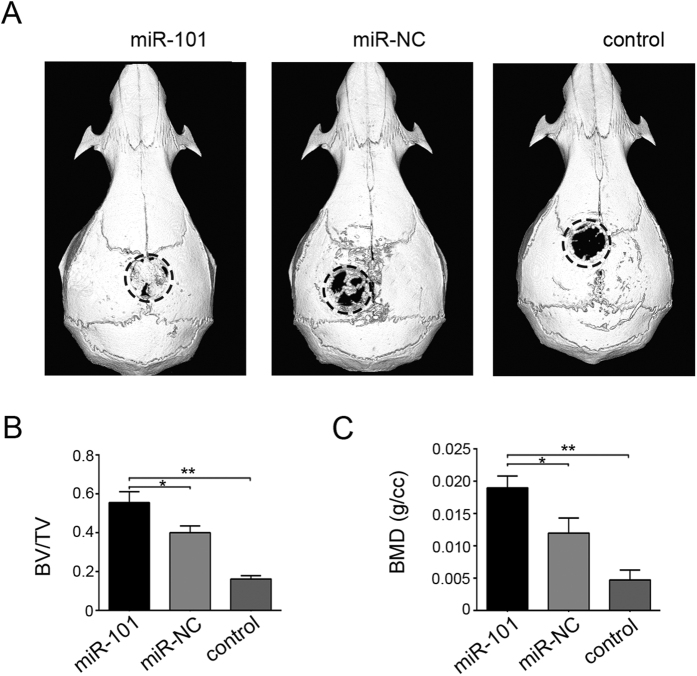
3-D reconstructed images of calvarial bone repair at week 8. (**A**) Representative images from coronal. (**B**,**C**) the bone volume/total volume (BV/TV) and bone mineral density (BMD) were analyzed, respectively. All of the data are expressed as the mean ± SD. *p < 0.05, **p < 0.01.

To investigate whether miR-101 regulated *in vivo* bone formation by hBMSCs, the compounds (the control (PseD alone), miR-101 (PSeD with miR-101-hBMSCs) and miR-101 (PSeD with miR-NC-hBMSCs) were established and implanted into the critical size nude mice calvarial defects model. 3-D reconstructed images of calvarial bone repair at week 8 was shown in Fig. 6A. Quantification of the bone volume fraction (bone volume/total volume; BV/TV) and bone mineral density (BMD) in each group was used to evaluate the calvarial bone repair regulated by miR-101. Fig. 6B showed that bone volume fraction (BV/TV) in miR-101 group was higher than miR-NC group and control group. Consistent with the above results, BMD of newly formed bone in miR-101 group was higher than miR-NC group and control group. These results suggested that miR-101 promotes *in vivo* bone formation by hBMSCs.

## Discussion

The differentiation of hMSCs, especially osteogenic differentiation, is precisely regulated by intracellular or extracellular mechanical and molecular signals and is a promising target for stem cell-based therapies for various human diseases^19^. However, the regulatory mechanisms of MSC fate determination remain poorly understood. Recently, merging evidence indicate that miRNAs play crucial roles in the osteogenic differentiation of MSCs through targeting the 3′-UTR of the essential transcription factors for osteogenic differentiation^20^. For instance, miR-346, which is upregulated during the osteoblast differentiation of hBMSCs, promotes osteogenic differentiation by targeting GSK-3β and regulating the Wnt/β-catenin differentiation signaling pathway^21^. Mizuno *et al.* reported that miR-210 promotes osteogenic differentiation by inhibiting the TGF-β/activin signaling pathway via the suppression of the expression of activin A receptor type 1B (AcvR1b)^22^. In contrast, miR-138 acts as negative regulator ofthe osteogenic differentiation of hBMSCs by targeting FAK and suppressing theFAK-ERK1/2 signaling pathway^7^,^23^. MiR-133 and miR-135 also negatively regulate the osteogenic differentiation of hBMSCs induced by BMP2 by directly targeting RUNX2 and Smad5, respectively^24^. In addition to these miRNAs, several other miRNAs negatively or positively modulate the osteogenic differentiation of hBMSCs.

Here, our results showed that miR-101 expression gradually increased during the osteogenic differentiation of hBMSCs, suggesting that miR-101 was involved in regulating this complex process. We next investigated the biological role of miR-101 in the osteogenic differentiation of hBMSCs. MiR-101 silencing significantly reduced osteoblastic differentiation, which was indicated by lower expression levels of the osteoblastic marker genes RUNX2, ALP, OPN and OCN, as well as decreased ALP activity and matrix mineralization levels; miR-101 overexpression was sufficient to promote this complex process. MiR-101, which functions as a tumor suppressor, has been shown to be down-regulated in several cancers and suppresses cancer cell proliferation, migration and invasion^12^,^13^,^15^, while functional studies in our experiments demonstrated that miR-101 is up-regulated and serves as a positive regulator in the osteogenesis of hBMSCs. One possible explanation for these discrepancies is that the decision of cells to initiate differentiation requires the inhibition of cell proliferation and cell cycle arrest^25^.

EZH2, acatalytic subunit of Polycomb repressive complex 2, has been reported to be involved in various biological processes including cell growth, differentiation and apoptosis^26^. Recent studies are beginning to unravel its importance in regulating osteogenic differentiation. Histone H3 lysine 27 (H3K27) methylation resulting from transcriptional repression regulated by EZH2 is responsible for this complexprocess^27^. Lu *et al.*^28^ demonstrated that EZH2 could suppress the hepatocellular differentiation of mouse MSCs by repressing the expression of AFP and FOXA2. The neuronal differentiation of mesenchymal stem cells was also inhibited by EZH2 through negatively mediating PIP5K1C-dependent calcium signaling^29^. In addition, the activity of cyclin-dependent kinase 1 promoted the osteogenic differentiation of hMSCs through the phosphorylation of EZH2 at Thr 487, resulting in the failure of EZH2-mediated H3K27 methylation^30^. Our results suggest that EZH2 is a target gene of miR-101; the expression of EZH2 was gradually down-regulated during osteogenic differentiation induction, and enforced expression of EZH2 could inhibit osteogenic differentiation^31^, demonstrating that EZH2is required for the promoting effect of miR-101 in regulating the osteogenic differentiation of hBMSCs.

It is generally accepted that the Wnt/β-catenin (canonical) pathway is essential for the commitment of osteoprecursors and important for the regulation of osteogenic differentiation. In more detail, the Wnt/β-catenin axis regulates osteoblast proliferation, maturation and mineralization^32^. Activation of the Wnt/β-catenin pathway by Wnt genes or other chemicals causes an accumulation of β-catenin in the cytoplasm and its eventual translocation into the nucleus to activate Wnt/β-catenin-responsive genes, such as c-Myc, CyclinD1, TCF-1 and LEF-1, to regulate various developmental processes, including osteogenic differentiation^33^,^34^. EZH2, an upstream regulator of Wnt/β-catenin signaling, could repress the expression of Wnt genes, including Wnt1,6,10 and 2b,in the cytoplasm, resulting in the inactivation of the Wnt/β-catenin pathwaybyinducingH3K27 methylation^17^. However, some researchers have reported that EZH2 is highly expressed in various cancers, and its ectopic expression could promote tumorigenesis by activating the Wnt/β-catenin pathway^35^,^36^. The probable reason for this difference is that EZH2 represses the expression of Wnt antagonists to activate the Wnt/β-catenin pathway in aH3K27 methylation-independentfashion^35^,^36^,^37^. In our present study, we demonstrate that the activity of the Wnt/β-cateninaxis plays an essential role in the triggering of osteogenic differentiation by miR-101. However, Lin Zhu *et al.*^4^ also found that ectopic EZH2 inhibited the osteogenic differentiation of the human fetal osteoblastic cell line hFOB1.19 by down-regulating Runx2 expression via catalyzing H3K27me3 in the Runx2 gene promoter. Thus, miR-101 promotes osteogenic differentiation at least partly through the activity of the Wnt/β-cateninaxis.

**Figure 7:**
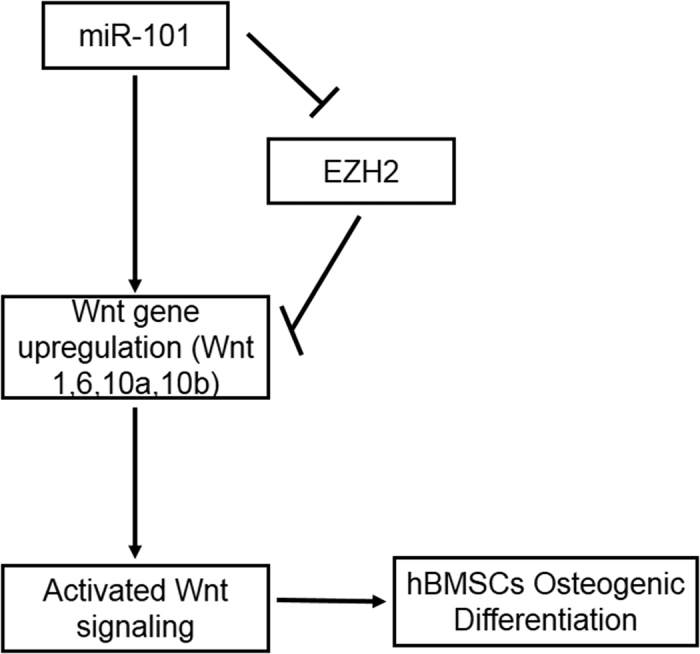
Model for how miRNA-101 facilitates osteogenic differentiation of hBMSCs. miR-101 promotes the osteogenic differentiation of hBMSCs by targeting EZH2, which results in the activation of the Wnt/β-catenin signaling pathway.

Overall, our data demonstrate that miR-101 promotes the osteogenic differentiation of hBMSCs by targeting EZH2, which results in the activation of the Wnt/β-catenin signaling pathway (Fig. 7). Our results provide new insight indicating that miR-101 possesses great potential as a novel class of therapeutic targets for bone regeneration.

## Materials and Methods

### Ethics statement

The animal studies were approved by the Animal Ethics Committees of Second Military Medical University. All animals were handled strictly according to the Good Animal Practice requirements of the Animal Ethics Procedures and Guidelines of the People’s Republic of China.

### Cell culture

Human mesenchymal stem cells (hBMSCs) were purchased from Cyagen Biosciences Inc (HUXMA-01001, China) and cultured in ORICell^TM^ Human Mesenchymal Stem Cell Growth Medium (cyagen Biosciences Inc, HUXMA-9011, China). HBMSCs from passage 3 to passage 5 were utilized for the osteogenic differentiation protocol. Briefly, hBMSCs were cultured in Human Mesenchymal Stem Cell Growth Medium at 37 °C in a 5% CO_2_ humidified incubator. When the cells were approximately 80–90% confluent, they could be dissociated and re-plated in growth medium at 1 × 10^5^cellsin 6-well tissue culture plates pre-coated with gelatin solution. After 24 h, the growth medium from each well was carefully aspirated off and 2 ml Human Mesenchymal Stem Cell Osteogenic Differentiation Medium (DMEM containing 10% FBS, 100 U/mL Penicillin-Streptomycin, 1% Glutamine, 10 nM dexamethasone,0.2 mM L-ascorbic acid, and 10 mM β-Mglycerophosphate) (Cyagen Biosciences Inc, HUXMA-90021,China)was added to induce osteogenic differentiation. The cells were cultured in differentiation medium for approximately 15 days with a medium change every3 days. The effects of ICG-001 (Selleck, USA) on the osteogenic differentiation of hMSCs have been described^21^. The cells were then cultured for approximately 15 days with differentiation media changed every 3 days with or without the presence ofICG-001.

### RNA preparation and quantitative real-time PCR (qRT-PCR)

Trizol reagent (Life Technologies, USA) and a miRNeasy Mini Kit (Qiagen, Germany) was used to isolate total RNA according to the manufacturer’s protocol as previously described^38^,^39^,^40^. Next, cDNA was synthesized by Reverse transcriptionand qPCR was carried out using themiScript SYBR Green PCR Kit (Life Technologies, USA)and themiScript/QuantiTect Peimer assay (Qiagen, Germany). The primer sequences for the miR-101 and U6^16^, Wnt1,6,10a and 10b^17^, RUNX2,ALP,OPN,OCN and β-actin and^41^, EZH2andβ-catenin^42^ isoforms were previously described. The expression levels of the miRNAs and genes are presented as values normalized against theU6 and n-actin transcripts, respectively. All samples were performed in triplicates.

### Alkaline phosphatase (ALP) assays

ALP enzyme activity was measured using an alkaline phosphatase detection kit (Jiancheng Bioengineering, China) based on the manufacturer’s protocol. In brief, cultured cells were washed with PBS and cracked with a solution containing 20 mMTris-HCl (pH 8.0), 150 mMNaCl, 1% Triton X-100, 0.02% NaN_3_and 1Tritonaprotinin. The lysates were homogenized using a pipette, incubated for 15 minutes at 37 °C, and assayed for ALP activity using a spectrophotometer. The results were normalized to the total cellular protein content.

### Alizarin Red Staining

The matrix mineralization was measured by Alizarin Red staining (Sigma, USA). Briefly, the cells were fixed with 70% ethanol for 1 h and rinsed with PBS, and then treated with 40 mM ARS solution at pH 4.2 for 10 min. After washing with phosphate-buffered saline for 15 min, the stained cells were photographed. For quantification of staining, the Alizarin Red S stain was released from the cell matrix by incubation in cetyl-pyridinium chloride for 15 min and the amount of released dye was measured by spectrophotometry at 540 nm. The results were normalized to the total cellular protein content.

### Lentivirus infection and oligonucleotide transfection

The miR-101, anti-miR-101 and EZH2 siRNA were purchased from Origene (Beijing, China). The constructs containing the pre-miR-101 or EZH2 siRNA sequences were cloned into the lentivirus-based expression plasmid pLenti-6.3 (Invitrogen, USA). The lentivirus was packaged and the positive clones were identified by Shanghai GenePharma Co., Ltd (China). HBMSCs (1 × 10^5^) were infected with 1 × 10^7^ recombinant lentivirus-transducing units plus 8 ug/mL Polybrene (Sigma, USA). An empty lentiviral vector was used as a negative control. To silence mirR-101 in hBMSCs, the cells were transfected with anti-miR-101 or anti-NC using Lipofectamin2000 reagent (Invitrogen, USA). The cells were collected for assays 48 hours after transfection.

### Plasmid construction

The full-length open reading frame of EZH2 was cloned into pcDNA3.1 (+) to generate expression vectors. The wild-type EZH23′-UTR was cloned into the pGL3-basic vector (Promega Corporation, USA). Site-directed mutagenesis of the miR-101 seed sequence in the 3′-UTR (Mut) was performed using the QuikChange™ Site-Directed Mutagenesis Kit (Stratagene, USA).

### Luciferase reporter assays

Light switch luciferase assay reagents were obtained from Promega (Corporation, USA). miR-101overexpressing hBMSCs were transfected together with a luciferase reporter plasmid and the pRL-TK vector expressing Renilla luciferase for 24 h. Luciferase activity was measured using a dual reporter assay system according to the manufacturer’s instructions. Renilla luciferase was used for normalization.

### TOPflash/FOPflash reporter assay

To test Wnt signaling, miR-101 overexpressing cells were co-transfected with either the Wnt signaling reporter TOPFlash or the negative control FOPFlash according to the protocol (Millipore, USA). Briefly, 1 × 10^4^ cells were transiently transfected with either 2 μg of pTOPflash (TCF reporter plasmid) or pFOPflash (mutant, inactive TCF binding site) plasmids (Millipore, USA) and 0.5 μg of pSV40-Renilla plasmid as an internal control (Promega Corporation, USA) using Lipo-2000 for 48 h. Both Firefly and Renilla luciferase activities were measured with a Glomax luminometer (Promega Corporation, USA) using the Dual-Glo Luciferase Assay System (Promega Corporation, USA).

### Western blot analysis

Cells were harvested in cell lysis buffer (Beyotime, China), and the protein content was quantified by BCA methods. The protein was separated onSDS-PAGE and transferred onto a nitrocellulose membrane. The membrane was incubated with the primary anti-EZH2, anti-β-actin and anti-β-catenin (Abcam, UK), anti-c-Myc, anti-CyclinD1, anti-TCF-1, anti-LEF-1 (Santa Cruz, USA), and active anti-β-Catenin (Millipore, USA) and secondary horseradish peroxidase-conjugated antibody. β-actin was used as an internal control.

### Surgical procedures

Surgical procedures were performed on immunocompromised nude mice (8-week-old, female, Slaccas Laboratory Inc., Shanghai, China) according to a previously reported method^43^. Briefly, the mice were anesthetized by intra-peritoneal injection of pentobarbital (Nembutal, 3.5 mg/100 g). Parietal bone was exposed by a 2 cm sagittal incision. The critical-sized calvarial defect (CSD)(4 mm in diameter) was created in the middle of parietal bone using a sterile drill. The constructs (PSeD with transduce hBMScs or empty PSeD scaffold) were implanted into the defects and the incision was closed.

### μ CT imaging

μ CT was performed using CT scanner (GE Healthcare Bio-Sciences Corp., Piscataway, NJ, USA). At 8 weeks post-transplantation, the specimens were harvested and scanned with 8.96th. Microstructural indices were measured using MicroView (GE Healthcare Bio-Sciences Corp., Piscataway, NJ, USA). The parameters of bone volume/tissue volume (BV/TV), trabecular thickness (Tb. Th), and bone mineral density (BMD)were calculated in this study.

### Statistical analysis

All experiments were performed in triplicates on the same sample and independently repeated three times. The data are expressed as mean ± SD. Statistical analyses were performed using Student’s T test or a one-way ANOVA for experiments with more than two subgroups. P < 0.05 was considered statistically significant.

## Additional Information

**How to cite this article**: Wang, H. *et al.* MiR-101 Targets the EZH2/Wnt/β-Catenin the Pathway to Promote the Osteogenic Differentiation of Human Bone Marrow-Derived Mesenchymal Stem Cells. *Sci. Rep.* **6**, 36988; doi: 10.1038/srep36988 (2016).

**Publisher’s note**: Springer Nature remains neutral with regard to jurisdictional claims in published maps and institutional affiliations.
